# OPTIC FLOW IMPROVES SPATIAL GAIT FUNCTIONING ESPECIALLY IN MEDIOLATERAL DIRECTION

**DOI:** 10.1093/geroni/igz038.2439

**Published:** 2019-11-08

**Authors:** Farahnaz Fallahtafti, Hyeon Jung Kim, Jennifer M Yentes, Dawn Venema, Julie Blaskewicz Boron

**Affiliations:** 1 University of Nebraska at Omaha, Omaha, Nebraska, United States; 2 University of Nebraska Medical Center, Omaha, Nebraska, United States

## Abstract

Instances where multiple tasks are completed simultaneously are considered high cognitive load situations (HCLS, also called dual-task), potentially affecting gait performance in older adults. Walking while talking is a common HCLS that requires additional cognitive resources. Optic flow (OF) provides visual information about speed and direction of self-motion, and thus, may ameliorate gait deficits under HCLS. This study aimed to identify the effect of HCLS, as well as OF, on gait performance in older adults. The HCLS included walking while talking on the phone, compared to walking alone. Fifteen older adults (70.86±4.7yrs) underwent four experimental conditions: walking alone with(1) and without OF(2), as well as walking while talking with(3) and without OF(4). Step width, step length, and double support time were measured and examined with 2(HCLS) x 2(OF) repeated-measures ANOVAs. There was a main effect of OF; step width was narrower with OF compared to without OF (p=0.048). For step length, there was a significant interaction between HCLS and OF (p=0.045). Without OF, there were no differences in step length; however, with OF step length was significantly longer when walking alone compared to when walking while talking (p=0.002). Double support time was not affected by HCLS or OF. Considering younger adults have longer and narrower steps compared to older adults, OF may have enhanced step width regardless of HCLS and step length when walking only. Using OF in training programs designed for older adults, could be a potential factor to improve spatial gait function, more so in the mediolateral direction.

